# Search for new steroidal glycosides with anti-cancer potential from natural resources

**DOI:** 10.1007/s11418-024-01830-1

**Published:** 2024-07-17

**Authors:** Yukiko Matsuo, Yoshihiro Mimaki

**Affiliations:** School of Pharmacy, Tokyo University of Pharmacy and Life Sciences, 1432-1, Horinouchi, Hachioji, Tokyo 192-0392 Japan

**Keywords:** Pregnane glycosides, Cardenolide glycosides, Spirostanol glycosides, Furostanol glycosides, Cytotoxic activity, Apoptosis

## Abstract

Chemical investigations of higher plants, with particular attention paid to their steroidal glycosides, present a promising approach for generating anti-cancer agents from natural products. We conducted a systematic phytochemical investigation of nine higher plants—whole plants and rhizomes of *Convallaria majalis*, whole plants of *Agave utahensis*, roots of *Adonis amurensis*, seeds of *Adonis aestivalis*, bulbs of *Bessera elegans*, bulbs of *Fritillaria meleagris*, seeds of *Digitalis purpurea*, underground parts of *Yucca glauca*, and bulbs of *Lilium pumilum—*which led to the discovery of novel steroidal glycosides. The structures of these new constituents were determined based on spectroscopic data and chemical transformations. The identification of the monosaccharides including their absolute configurations was carried out by direct HPLC analysis of their hydrolysates using an optical rotation detector. Cytotoxicity of the isolated steroidal glycosides was evaluated against various tumor cells (A549, ACHN, HepG-2, HL-60, HSC-2, HSC-3, HSC-4, HSG, and SBC-3) and normal cells (Fa2 N-4, HK-2, and TIG-3 cells). Certain steroidal glycosides exhibit selective cytotoxicity and synergistic effects, making them potential lead compounds for use as anti-cancer agents. We document the isolation of 139 steroidal glycosides from higher plants and assessment their cytotoxic activities.

**Graphical abstract:**

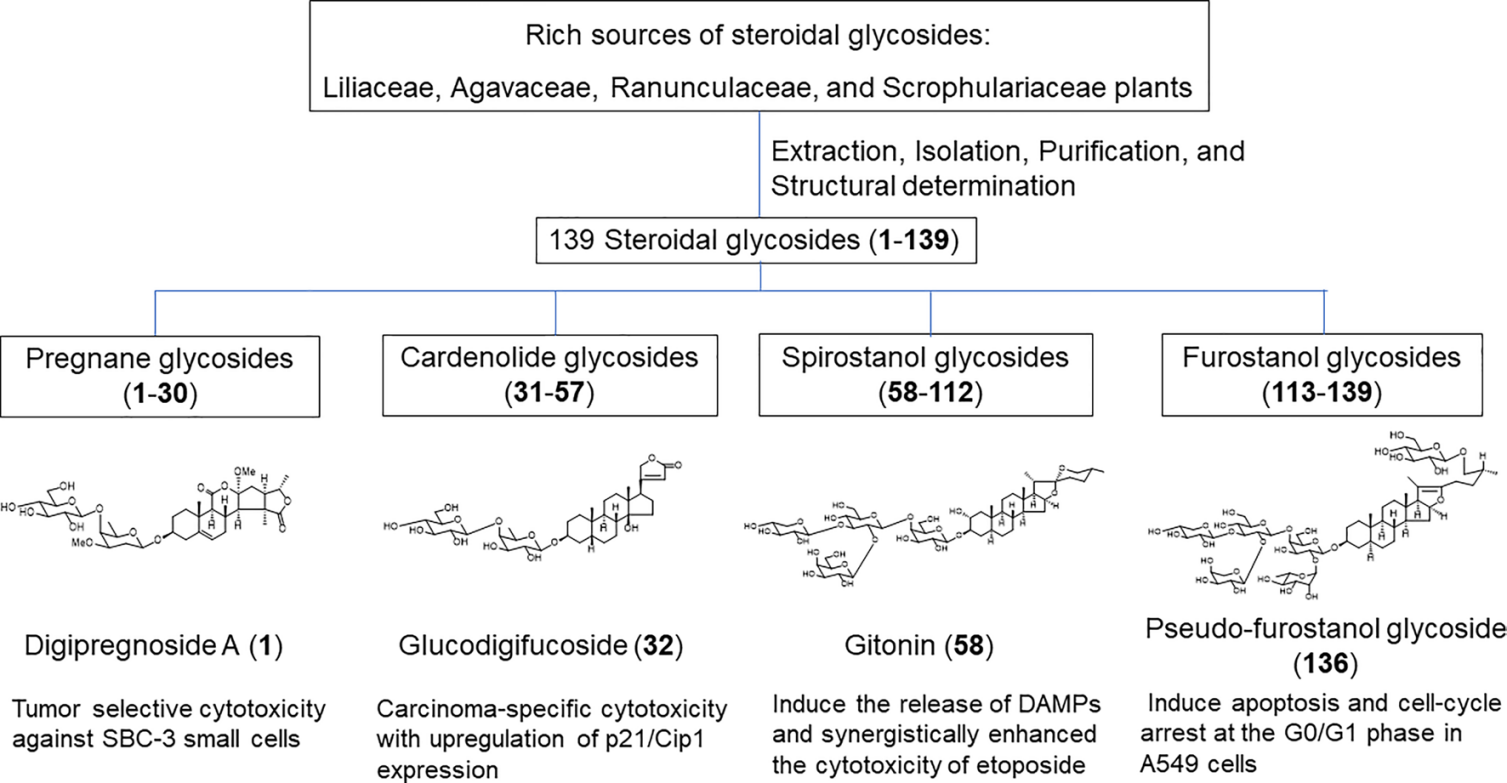

## Introduction

Steroidal glycosides constitute a group of natural products found in a limited range of plant families, including Agavaceae, Leguminosae, Liliaceae, Plantaginaceae, Ranunculaceae, and Solanaceae. Predominantly present as glycosides in nature, steroidal glycosides display a diverse array of chemical structures. These compounds are categorized as spirostanol, furostanol, pregnane, cardenolide, and cardiac glycosides, based on their aglycone structures. Furthermore, steroidal glycosides from higher plants have demonstrated various biological activities, including antitumor, antidiabetic, antitussive, and inhibition of platelet aggregation [1, 2]. In vitro studies have identified cytotoxic activities against tumor cell lines including A549, Caco-2, Hela, HepG-2, HL-60, HT-29, MCF-7, PC-3, SGC-7901, and U251, showing mechanisms, such as apoptosis, necrosis, autophagy, ferroptosis, and cell cycle arrest [3–6]. Terrestrosin D, a spirostanol glycoside isolated from *Tribulus terrestris*, has shown to induce cell cycle arrest in the G_1_ phase and stimulate the caspase-independent apoptosis of PC-3 cells [5]. A novel spirostanol glycoside T-17, isolated from *Tupistra chinensis*, induces apoptosis and triggers cytoprotective autophagy in human gastric cancer cell lines by activating the JNK pathway [7]. Thus, a large amount of cytotoxic data against a variety of tumor cells were performed and the molecular mechanism of steroidal glycosides was elucidated. Although in vivo experiments on their anti-cancer activities are limited, certain steroidal glycosides have been shown to exhibit anti-cancer effects. For instance, oleandrin (administered at 0.3 or 0.6 mg/kg, i.p., 7 days) suppressed tumor growth in mice bearing EMT6 murine mammary carcinoma [8]. In another study, cotreatment of glioma bearing mice with temozolomide (TMZ) and oleandrin (administered at 0.3 mg/kg, i.p., 100 days) strongly prolongs mice survival compared with TMZ treatment alone [9]. Garofalo et al. demonstrated the potential of oleandrin as a co-adjuvant drug in standard chemotherapeutics. Additionally, OSW-1, a steroidal cholestane diglycoside isolated from *Ornithogalum saundersiae*, significantly boosted the anti-metastatic ability of doxorubicin in 4T1 mice and enhanced CD8^+^ T cell infiltration into the immune microenvironment of the lungs [10].

Immune checkpoint inhibitors (ICIs) and molecular targeted therapeutic agents have emerged as promising tools for cancer therapy. However, resistance to ICIs can develop in some patients, leading to a lack of an objective response [11]. Between 1981 and 2019, 25% of all the anti-cancer drugs approved by the Food and Drug Administration were derived from natural products or natural product derivatives [12], highlighting the potential of novel anti-cancer agents in chemotherapy. Steroidal glycosides might be considered as lead compounds for preclinical studies in cancer chemotherapy.

This review presents our previous phytochemical studies on steroidal glycosides from nine plant sources: the whole plants and rhizomes of *Convallaria majalis* (Liliaceae) [13–15], whole plants of *Agave utahensis* (Agavaceae) [16–18], roots of *Adonis amurensis* (Ranunculaceae) [19, 20], seeds of *Adonis aestivalis* (Ranunculaceae) [21, 22], bulbs of *Bessera elegans* (Liliaceae) [23], bulbs of *Fritillaria meleagris* (Liliaceae) [24], seeds of *Digitalis purpurea* (Scrophulariaceae) [25–28], underground parts of *Yucca glauca* (Agavaceae)[29], and bulbs of *Lilium pumilum* (Liliaceae) [30]. These studies also investigated the cytotoxic activities of the isolated compounds against tumor cells and normal cells. Plants belonging to the families Liliaceae and Ranunculaceae are particularly rich sources of bioactive steroidal glycosides, such as OSW-1 or galtonioside A, which exhibit cytotoxic activity against tumor cells [31–33]. Furthermore, potent cytotoxic compounds have been discovered in popular ornamental garden plants, such as *Bessera elegans* and *Fritillaria meleagris* [23, 24]. These results indicate that garden plants with no proven medicinal backgrounds may also be potential sources of novel drugs.

## Pregnane glycosides

Pregnane glycosides, initially isolated from *Digitalis purpurea* and termed “digitanol glycosides”, are now found in various Apocynaceae, Asclepiadaceae, Malpighiaceae, Ranunculaceae, and Zygophyllaceae species [34]. Structurally, pregnane glycosides consist of six-membered A–C rings and a five-membered D ring in the C21 steroid skeleton, commonly featuring deoxy sugars linked to C-3 of the aglycone. The C/D *cis* structure of pregnane glycoside was synthesized via the cardenolide route. Based on the substituents and degree of oxidative cracking of the aglycone ring, the skeleton of the aglycone structure is divided into diverse structures. Various types of pregnane skeletons show various biological activities, including antibacterial, antidiabetic, anti-inflammatory, anti-obesity, and antitumor activities [34].

**Fig. 1:**
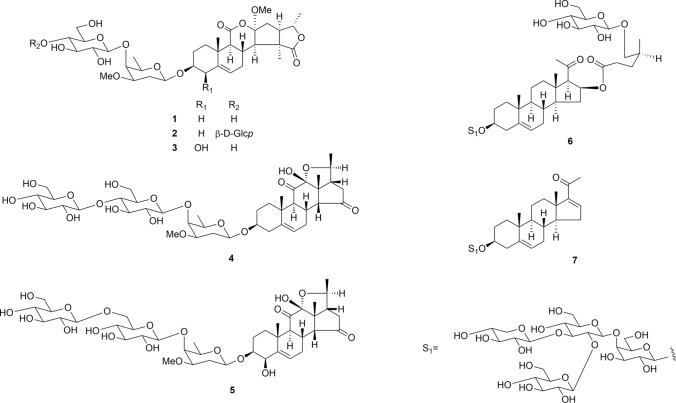
Structures of pregnane glycosides **1**–**5** isolated from the seeds of *Digitalis purpurea* and **6** and **7** from the whole plants of *Convallaria majalis*

**Fig. 2:**
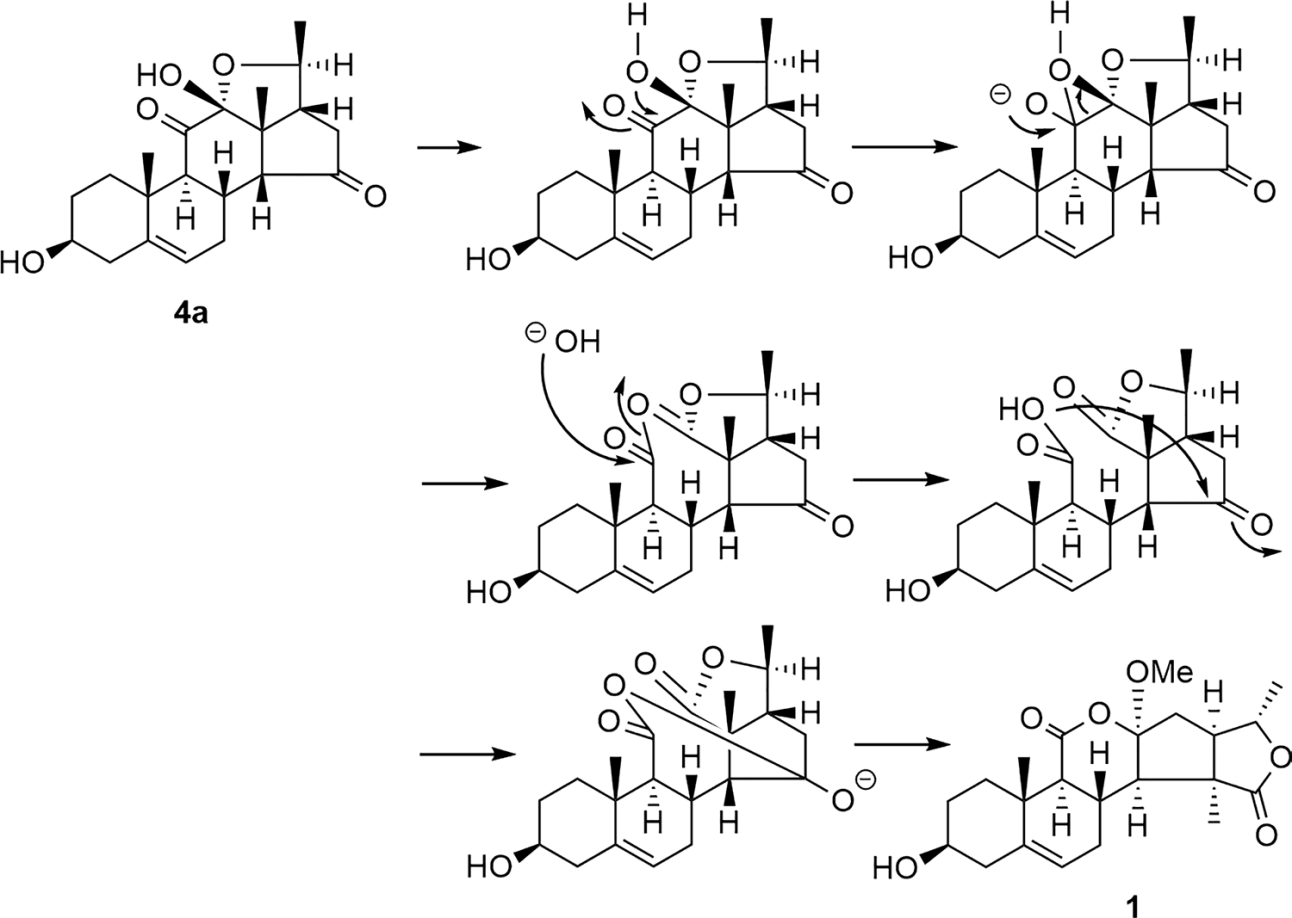
A possible biosynthetic pathway of the aglycone moiety of **1** from **4a**

From the seeds of *D. purpurea*, three novel rearranged 11,12-secopregnane glycosides, digipregnosides A (**1**), B (**2**), and C (**3**), and two novel 12,20-epoxypregnane glycosides, digipregnosides D (**4**) and E (**5**), were isolated (Fig. 1) [28]. The chemical structures of **1**–**5** were determined based on extensive spectroscopic analyses, including long-range heteronuclear single quantum multiple bond correlation (HSQMBC) spectral data and hydrolytic cleavage results. The HSQMBC spectrum provides more than ^3^*J*_C,H_ coupling correlations and suggested a novel skeleton of **1**. A plausible biogenetic pathway of the novel rearranged 11,12-secopregnane skeleton is proposed (Fig. 2), wherein the aglycone moiety of **1** is presumed to be biosynthesized from that of **4** (4a). After the C-11/C-12 bond of ring C was oxidatively cleaved, the hydroxy group of the C-11 carboxy functionality attacks the C-15 carbonyl group, resulting in the formation of γ- and δ-lactone rings. Evaluation of **1** and **3**–**5** for their cytotoxic activities against SBC-3 small cell lung carcinoma cells and TIG-3 normal human fibroblast cells was performed using a modified 3-(4,5-dimethylthiazol-2-yl)-2,5-diphenyl-2*H*-tetrazolium bromide (MTT) assay. Compounds **1**, **4**, and **5** exhibited cytotoxic activity against SBC-3 cells, with IC_50_ values of 1.3, 0.17, and 2.1 μM, respectively. Notably, **1** and **4** showed tumor-selective cytotoxicity against SBC-3 cells at 0.001 μM, inducing apoptotic cell death through caspase-3/7 activation [28].

**Fig. 3:**
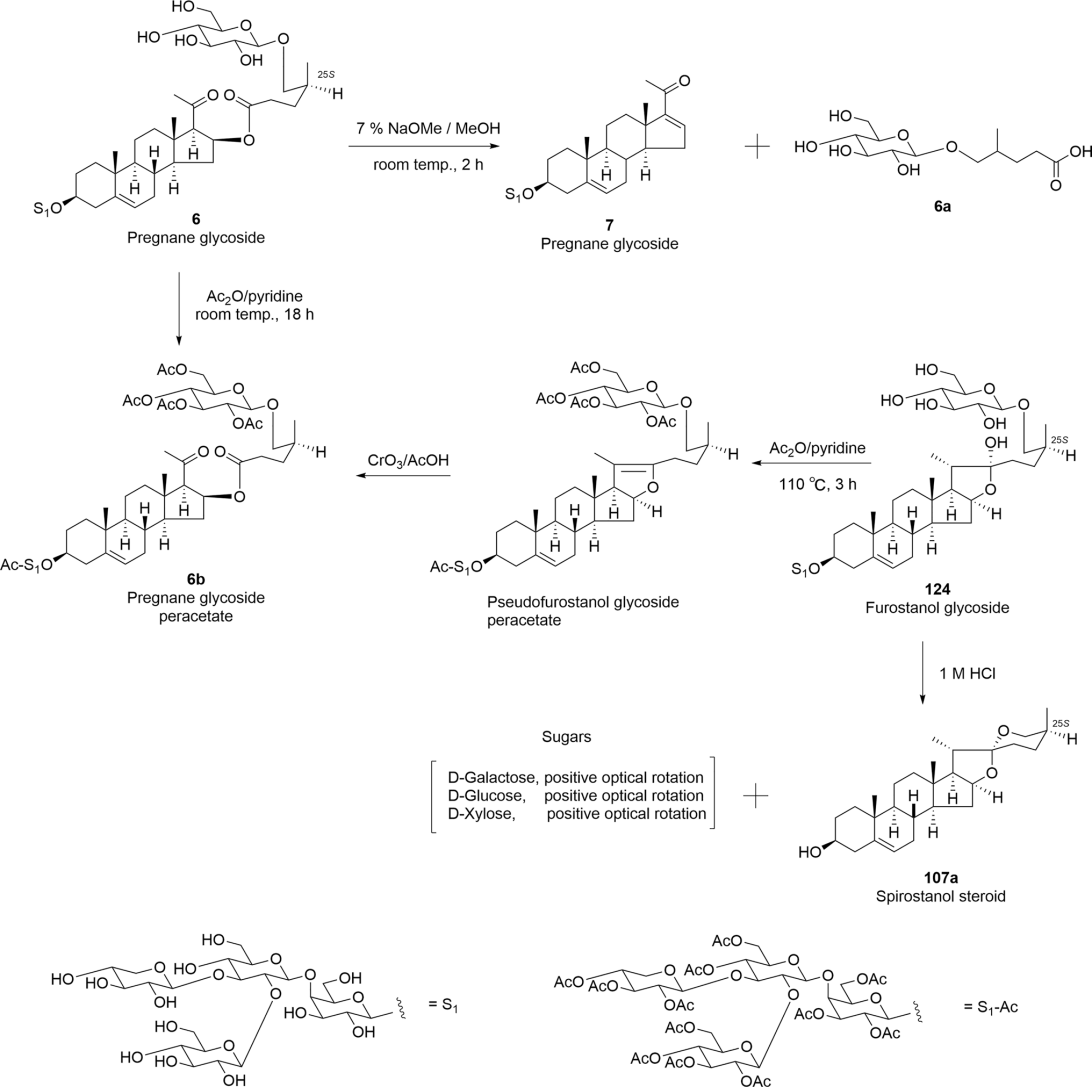
Chemical transformations of **6**

Chemical transformations were conducted to support the chemical structure determination of steroidal glycosides, including spirostanol, furostanol, pseudo-furostanol, and pregnane (Fig. 3). The new pregnane glycoside (**6**), isolated from the whole plants of *Convallaria majalis*, was formed from a pseudo-furostanol glycoside through the oxidative cleavage of the C-20(22) double bond. This was confirmed by the fact that the peracetate (**6b**) of **6** was identical to the product obtained by treating furostanol glycoside (**124**) with acetic anhydride (Ac_2_O) in pyridine at 110 ℃ for 3 h followed by treatment with CrO_3_ in acetic acid (AcOH). Compound **6** was subjected to alkaline methanolysis with 7% sodium methoxide to obtain pregnane glycoside (**7**) and 5-[(β-D-glucopyranosyl)oxy]-4-methyl pentanoic acid (**6a**). Conversely, acid hydrolysis of **124** with 1 M HCl yielded a spirostanol steroid (**107a**) as the aglycone and D-galactose, D-glucose, and D-xylose as carbohydrate moieties [14]. Identification of the monosaccharides was conducted by direct HPLC analysis of the hydrolysate using a combination of refractive index and optical rotation detector. Compounds **6** and **7**, isolated from the whole plants of *Convallaria majalis*, did not show cytotoxic activity against HL-60 human promyelocytic leukemia cells, A549 human lung adenocarcinoma cells, HSC-4, and HSC-2 human oral squamous cell carcinoma cells (IC_50_ > 12 μM).

**Fig. 4:**
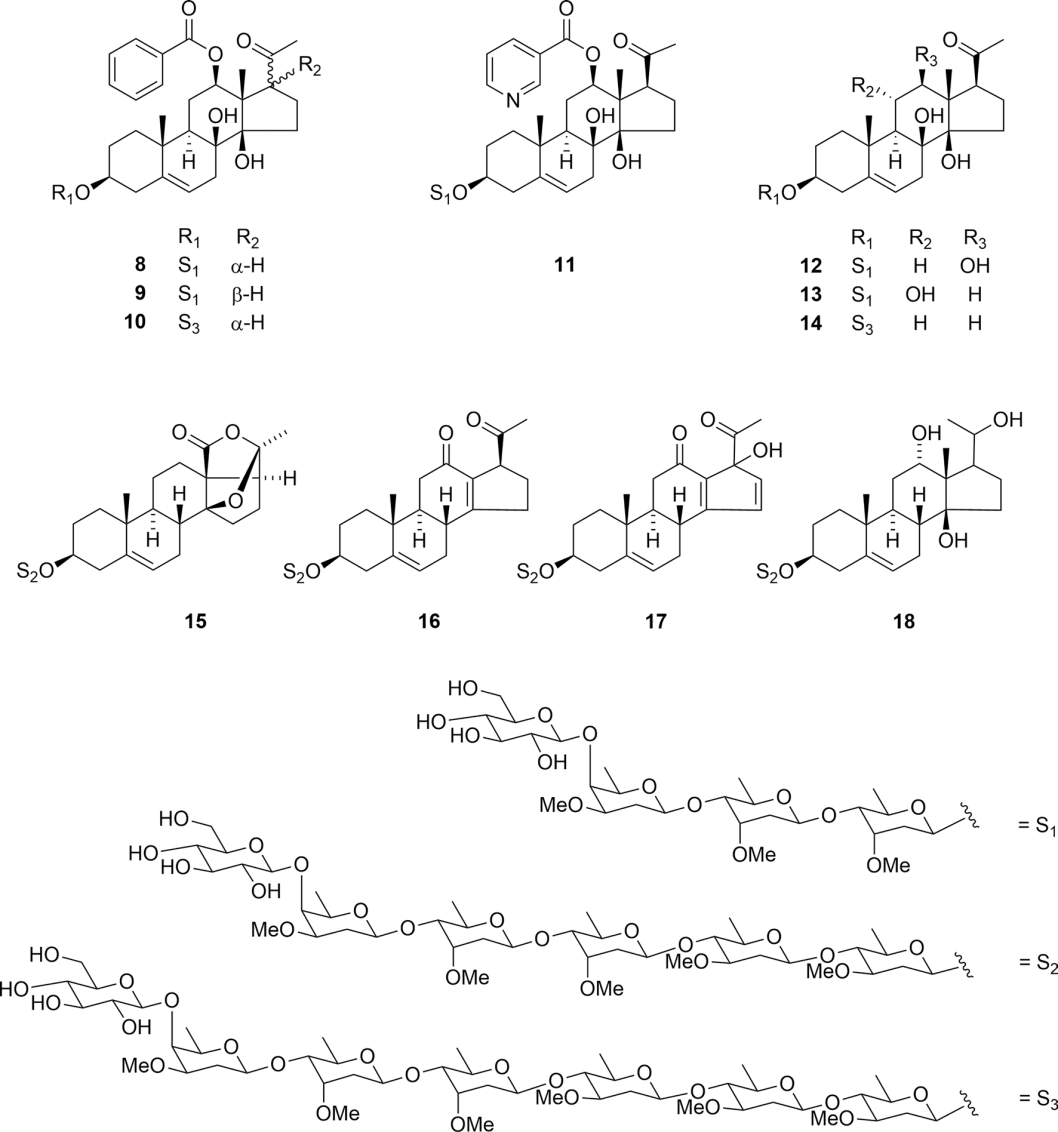
Structures of pregnane glycosides **8**–**18** isolated from the roots of *Adonis amurensis*

Amurensiosides A–K (**8**–**18**), isolated from the roots of *Adonis amurensis*, are newly discovered tetra-, hexa-, and heptaglycosides that contain deoxysugars (D-cymarose and D-diginose), characteristic pregnane and cardiotonic glycosides in plants (Fig. 4) [19]. The configuration of C-17 was confirmed via NOESY correlation between H-17 and Me-18. Aglycones of **13**, **17**, and **18** represent novel polyoxygenated pregnane derivatives. The aglycone of **15**, adonilide, is a hexacyclic pregnane featuring a five-membered lactone group and an ether linkage between C-14 and C-20. While the aglycones of **15** (adonilide) and **16** (fukujusonorone) were previously reported in the 1960s [35, 36], their glycosides remain unreported. Among **8**–**18**, compounds **8**, **9**, **12**, and **13** exhibited cytotoxicity solely toward HSC-2 cells (IC_50_ 66, 26, 47, and 58 μg/mL, respectively). This result indicates that replacing the benzoyl group with the nicotinoyl group at C-12 reduced the cytotoxic activity.

**Fig. 5:**
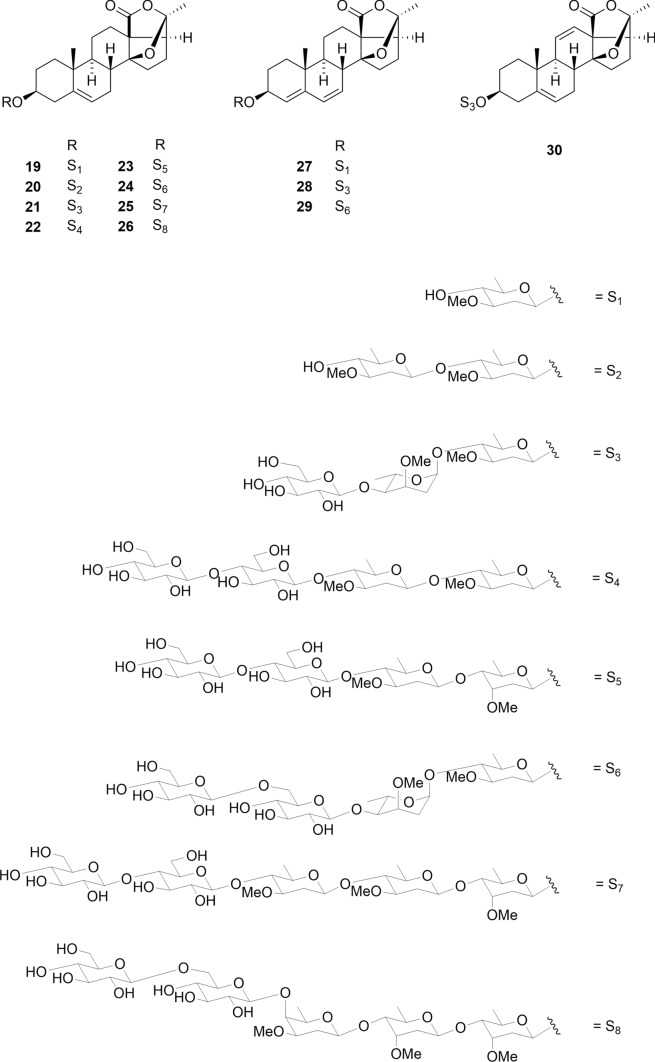
Structures of pregnane glycosides **19**–**30** from the seeds of *Adonis aestivalis*

The aglycones of Aestivalosides A-L (**19**–**30**) were isolated from the seeds of *Adonis aestivalis* and previously undescribed adonilide derivatives (Fig. 5) [22]. Oligoglycosides attached to C-3 of the aglycone of Aestivalosides B-H (**20**–**26**) are novel di-, tri-, tetra-, and pentaglycosides containing the following deoxysugars: D- and L-cymarose, D-diginose, and D-oleandrose. Adonilide derivatives have been previously identified in a limited number of *Adonis* species, including *A. amurensis* and *A. multiflora*. This is the first report of pregnane glycosides from *A. aestivalis* [37–39]. Compounds **19**–**30** were not cytotoxic to HSC-2, HSC-3, HSC-4, and HL-60 cells, even at sample concentration of 90 μM.

## Cardenolide glycosides

Cardenolide glycosides are a group of naturally occurring compounds found in several plant families including Apocynaceae, Liliaceae, Ranunculaceae, and Scrophulariaceae. Cardenolide has α,β-unsaturated butyrolactone ring at C-17 of the aglycone, a 14β-hydroxy group at C-14, and a C/D *cis* ring connection in the C23 steroid skeleton. The oligoglycosides attached to the C-3 of the aglycones comprise deoxysugars, which are typical of plant pregnane and cardiotonic glycosides. Cardenolide glycosides selectively inhibit Na^+^/K^+^-ATPase pumps. Recent studies demonstrated the specific cytotoxicity of cardenolide glycosides against renal adenocarcinoma and hepatocellular carcinoma cells, indicating their potential as candidates for anti-cancer drug development. Strophanthidin, a cardiac glycoside isolated from *Strophanthus kombe*, induces apoptosis by attenuating multiple biochemical signaling pathways and arresting the cell cycle at the G2/M phase through p53-dependent and -independent mechanism in A549, HepG-2, and MCF-7 cells [40]. Furthermore, strophanthidin inhibited autophagy by inhibiting the expression of the LC3 and p62 complexes in A549 cells [3]. Oleandrin, a cardiac glycoside isolated from *Nerium oleander*, induces apoptosis in SW480 human colorectal cancer cells via the mitochondrial pathway, without significantly reducing the viability of NCM460 normal human colonic epithelial cells [4].

**Fig. 6:**
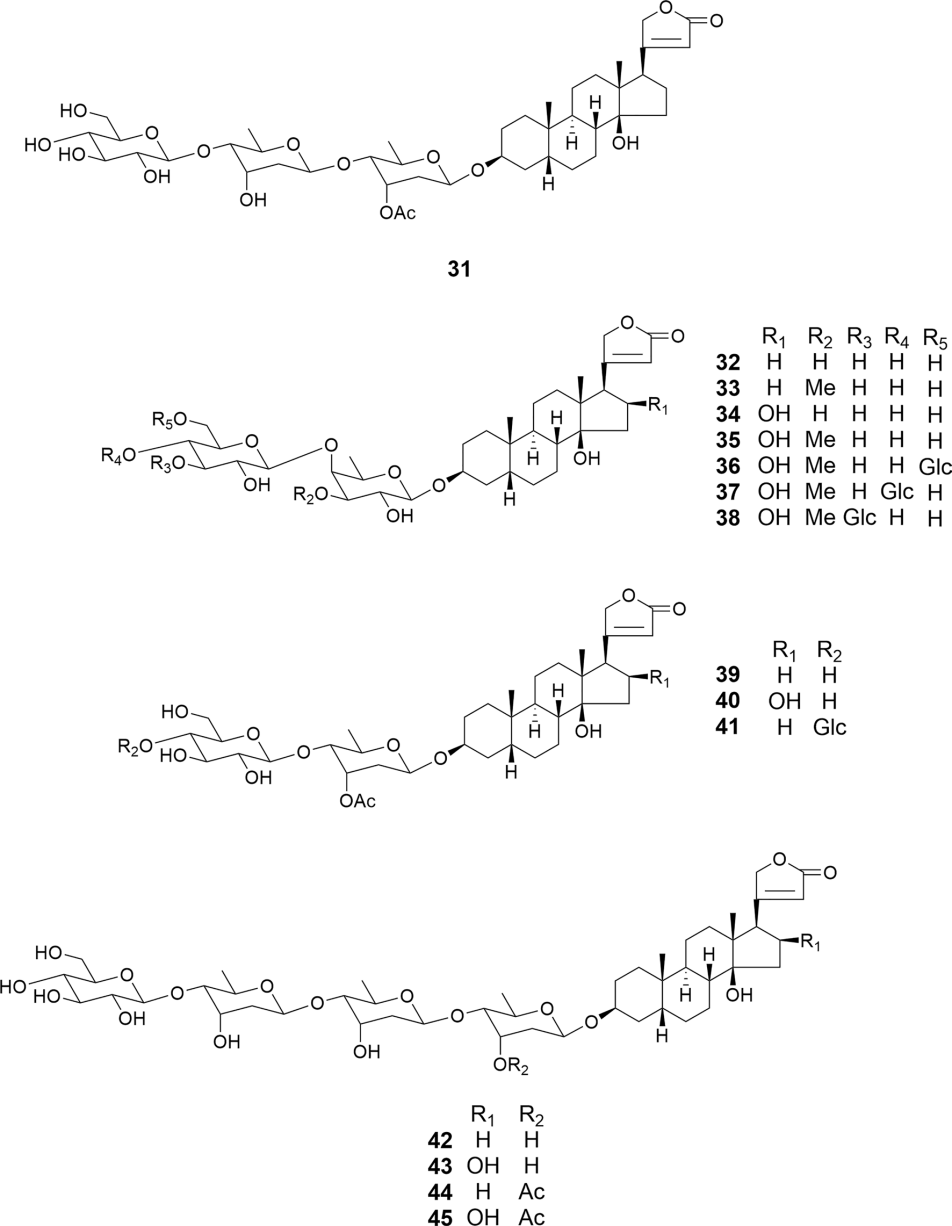
Structures of cardenolide glycosides **31**–**45** isolated from the seeds of *Digitalis purpurea*

**Fig. 7:**
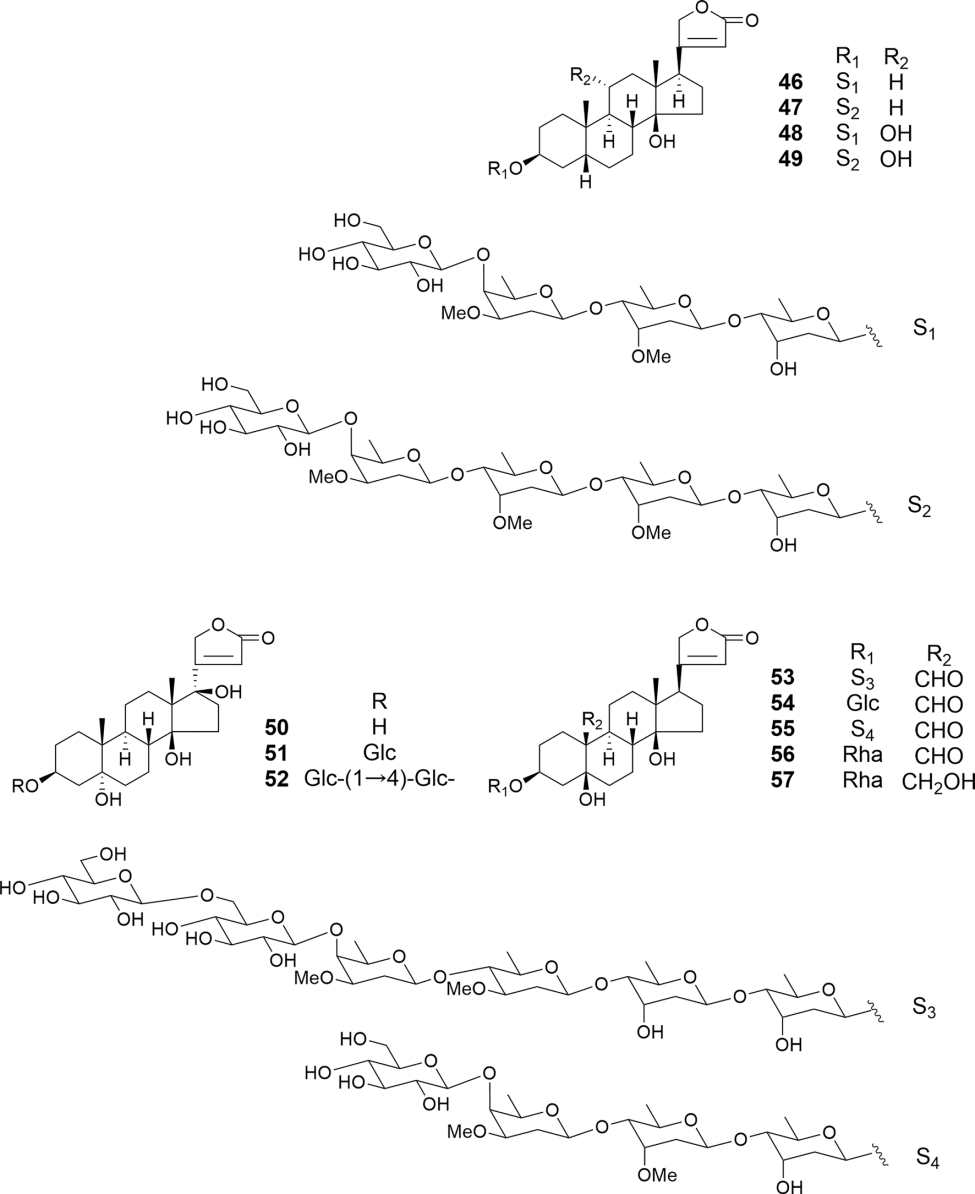
Structures of cardenolide glycosides **46**–**49** and **55** from the roots of *Adonis amurensis*, **50**–**54** from the seeds of *Adonis aestivalis*, and **56** and **57** from the rhizomes of *Convallaria majalis*

The structures of cardenolide glycosides (**31**–**45**), isolated from the seeds of *Digitalis purpurea* and including newly identified compounds (**31**, **38**, and **41**), contain digitoxigenin or gitoxigenin as the aglycone moiety, with D-glucose, D-digitoxose, and D-digitalose as sugar moieties (Fig. 6) [27]. Amurensiosides L-O (**46**–**49**), also newly discovered from the roots of *Adonis amurensis*, feature digitoxigenin as the aglycone, along with D-glucose, D-digitoxose, D-diginose, and D-cymarose as sugar moieties (Fig. 7) [20]. Among **31**–**49**, compounds **32**, **39**, **41**, **42**, and **46**–**49** exhibit potent cytotoxic activity against HL-60 cells (IC_50_ 0.034–0.069 μM). While the introduction of a hydroxy group at the C-16 position of the digitoxigenin aglycone reduced its cytotoxic activity (gitoxigenin > digitoxigenin), hydroxylation at the C-11α position shows no influence on the activity. 3-*O*-Methylation of the (inner) C-3’ hydroxy group at the fucopyranosyl moiety and 3-*O*-acetylation of the (inner) C-3’ hydroxy group at the digitoxopyranosyl moiety reduced cytotoxic activity. Conversely, glycosylation of the terminal glucosyl moiety of **35** and **39** had little effect on activity. As expected from a previous study [41], a steroidal aglycone (digitoxigenin) displayed weaker cytotoxic activity (IC_50_ 0.22 μM) than corresponding glycoside 46 (IC_50_ 0.057 μM) against HL-60 cells. Additionally, **32**, **39**, **41**, and **42** exhibit greater cytotoxicity against ACHN human renal adenocarcinoma cells (IC_50_ 0.02, 0.18, 0.08, and 0.47 μM, respectively) compared to HK-2 human embryonic kidney cells (IC_50_ 0.16, 0.37, 0.24, and 0.76 μM, respectively) [25]. In addition, glucodigifucoside (**32**) demonstrated greater specificity against HepG-2 human liver cancer cells (IC_50_ 0.21 μM) compared to that of Fa2 N-4 (IC_50_ 1.25 μM). Notably, **32** did not induce typical apoptotic morphological changes or alter Bax or Bcl-2 expression levels in ACHN cells. Compound **32** exerted carcinoma-specific cytotoxicity via p53-induced p21/Cip1 expression, independent of the p53-evoked pro-apoptotic pathway. Compounds **46**–**49** (IC_50_ 0.21–1.5μM) and digitoxigenin (IC_50_ 0.35 μM) also showed potent cytotoxic activity against HSC-2 cells.

5α-Hydroxy cardenolides (**50**–**52**) and 5β-hydroxy cardenolides (**53** and **54**) were isolated from the seeds of *Adonis aestivalis* (Fig. 7) [21]. These cardenolides consist of cardenolide or strophanthidin as the aglycone and D-oleandrose, D-diginose, D-digitoxose, and D-glucose as the carbohydrate components. In the **50**–**52**, the 17β-hydroxy configuration was confirmed by the following NOE correlations: from Me-18 to the two hydroxy groups at C-14 and C-17/H-21β, respectively. Cardenolides **50**–**54** were assessed for their cytotoxic activity against HSC-2, HSC-3, and HSC-4 human oral squamous cell carcinoma and HL-60 cells, as well as against human non-malignant HGF gingival fibroblasts, HPLF periodontal ligament fibroblasts, and HPC pulp cells. Compounds **51**, **53**, and **54** exhibited potent cytotoxic activity against HSC-2, HSC-3, HSC-4, and HL-60 cells (IC_50_ 0.013–2.8) and demonstrated selective cytotoxicity toward malignant cells. Particularly, among the 5α-hydroxy cardenolides (**50**–**52**), only the monoglucoside (**51**) was cytotoxic to tumor cell lines. Cell death of HSC-2 and HL-60 caused by **51**, **53**, and **54** was partially mediated through the induction of apoptosis, along with observed nuclear chromatin condensation and cell shrinkage. However, the typical laddering of DNA fragmentation and activation of caspase-3 was not detected. Therefore, **51**, **53**, and **54** may trigger caspase-3 independent apoptotic cell death in HSC-2 and HL-60 cells.

Amurensioside P (**55**), which was isolated from the roots of *Adonis amurensis*, exhibited potent cytotoxic activity against HL-60 and HSC-2 cells (IC_50_ 0.048 and 0.22 μM), respectively (Fig. 7) [20]. Consistent with previous findings, its steroidal aglycone, strophanthidin, displayed weaker cytotoxic activities against HL-60 and HSC-2 cells (IC_50_ 0.19 and 0.32 μM).

Convallatoxin (**56**) and convallatoxol (**57**), which were isolated from the rhizomes of *Convallaria majalis*, displayed potent cytotoxic activity against HSG human submandibular gland carcinoma cells with IC_50_ values of 0.012 and 0.028 μg/mL, respectively (Fig. 7). However, at a sample concentration of 0.10μg/mL, they exhibited no cytotoxicity against normal HPLF human periodontal ligament fibroblasts cells [13].

## Spirostanol glycosides

The aglycones of spirostanol glycosides are divided into the 5α(A/B-*trans*), 5β(A/B-*cis*), and 5(6)-ene types with A and B rings. 5β-steroidal glycosides, a minority among naturally occurring steroidal glycosides compared to 5α- and 5(6)-ene steroidal glycosides, are exclusively found in a limited number of higher plants belonging to the Agavaceae and Liliaceae families [42–45].

### 5α-spirostanol glycosides

**Fig. 8:**
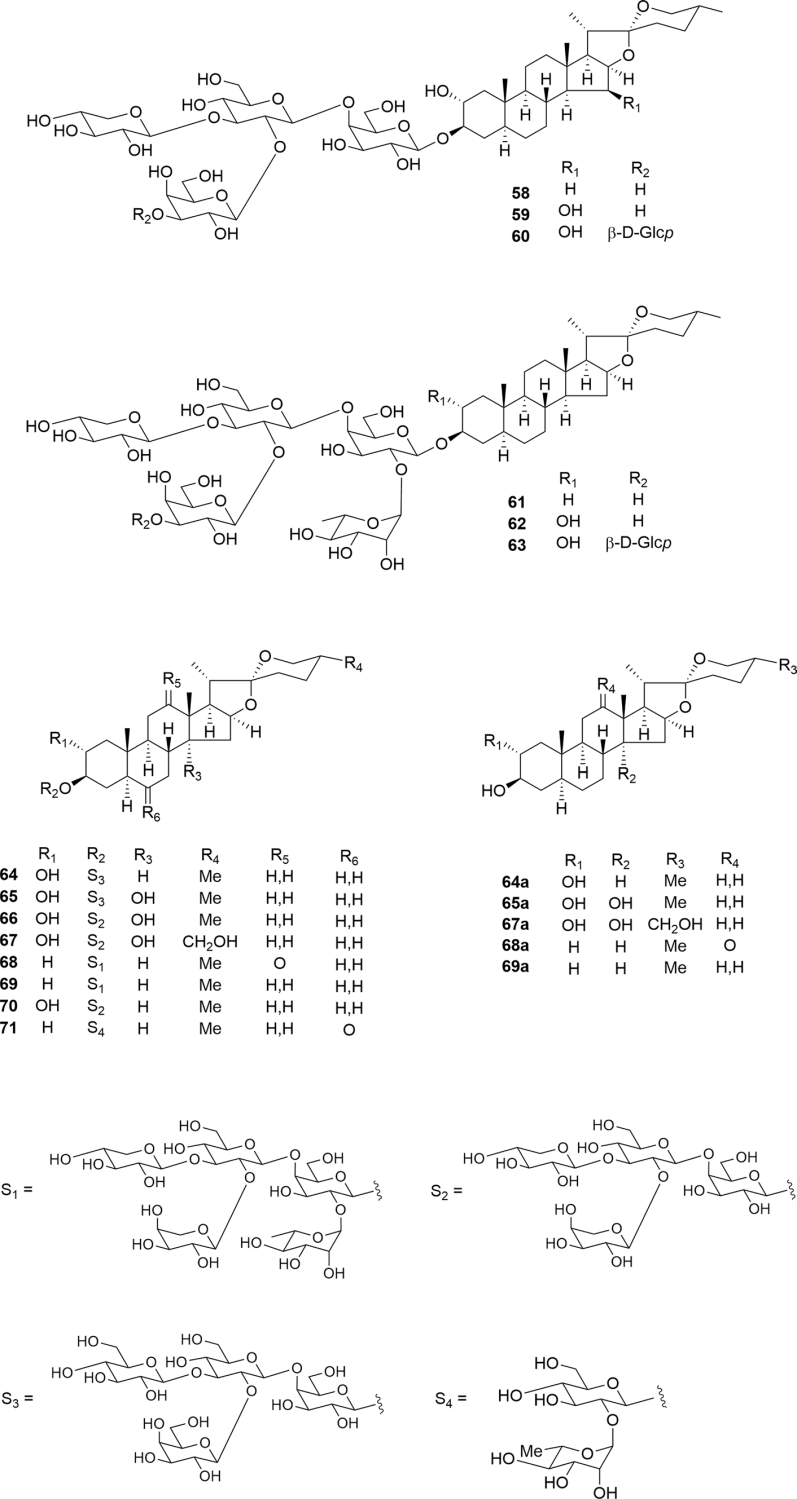
Structures of 5α-spirostanol glycosides **58**–**63** from the seeds of *Digitalis purpurea*, **64**–**70** isolated from the bulbs of *Bessera elegans*, and **71** from the bulbs of *Fritillaria meleagris*

**Fig. 9:**
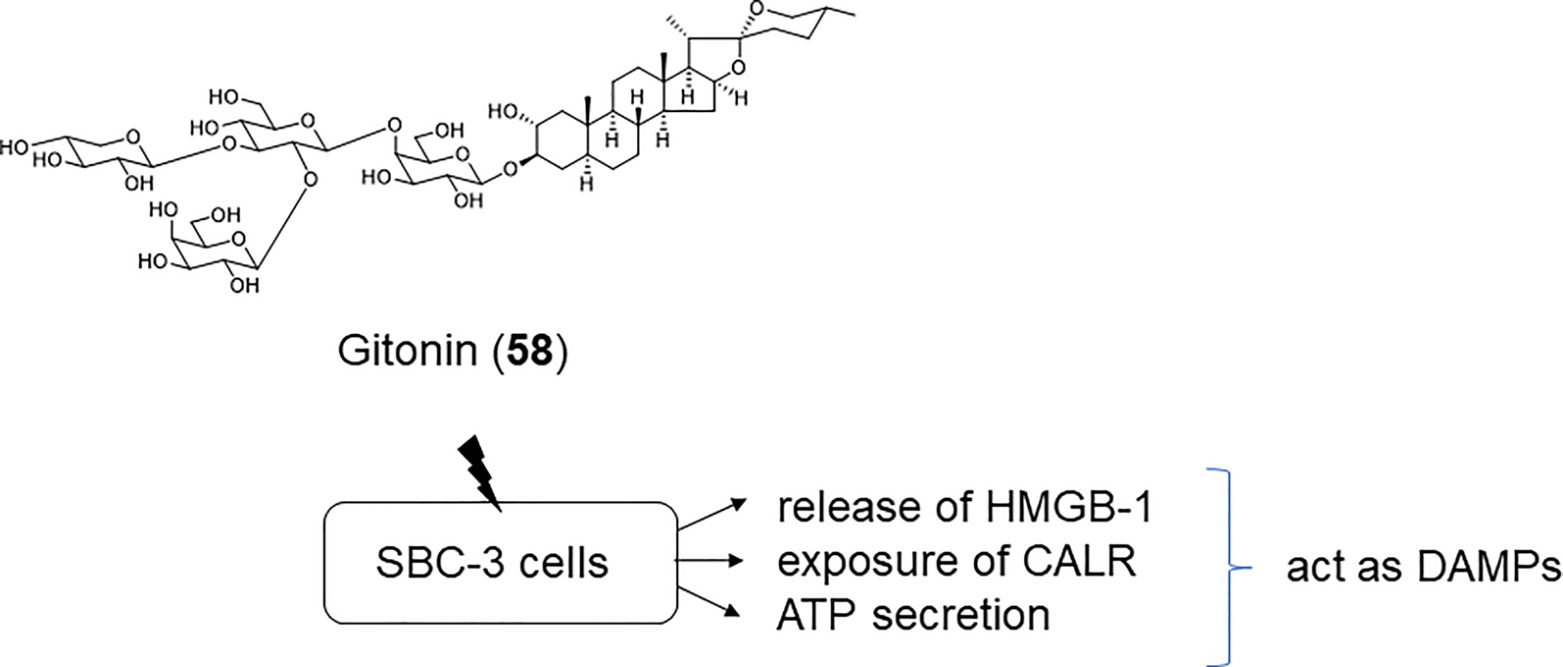
DAMPs-releasing activities of **58**

Among 5α-spirostanol glycosides **58**–**63**, which were isolated from the seeds of *Digitalis purpurea*, both tetra- and pentaglycosides (**58**–**62**) were cytotoxic to SBC-3 cells comparable to the positive control, etoposide (IC_50_ **58**–**62**: 1.0–1.7 μM; etoposide: 1.0 μM) (Fig. 8) [26]. Glucosylation at C-3 in the terminal galactopyranosyl moiety of **62** significantly diminished its cytotoxicity (IC_50_ **63**: > 10 μM). Compounds **60** and **62** showed high selectivity index (SI) values (> 5.9 and 10) between the SBC-3 tumor cells and TIG-3 normal cells. Compound **60** induced apoptotic cell death along with caspase-3 activation in SBC-3 cells, whereas **62** did not induce any apoptotic features in SBC-3 cells. Combining each of **58** and **60**–**62** (0.1 and 1.0 μM) with etoposide (0.01 and/or 0.1 μM) showed a synergistic effect (combination index (CI) values 0.17–0.50) against SBC-3 cells. Notably, the combination of gitonin (**58**) and etoposide achieved a strong synergistic effect (CI 0.27–0.43), although this synergistic effect was reduced by the introduction of a hydroxy group at the C-15 position (**59**). Among **58**–**63**, gitonin (**58**) notably released high mobility group box (HMGB)-1 in the preliminary screening assay. HMGB-1 is a DNA-binding protein in the nucleus; when released by dying cells, it acts as a damage-associated molecular pattern (DAMP) to trigger a strong inflammatory response [46]. Furthermore, the combination of **58** and etoposide resulted in an increase in the extracellular release of DAMPs, including the release of HMGB-1, secretion of ATP, and exposure of calreticulin (CALR) in SBC-3 cells. DAMPs can induce immunogenic cell death (ICD), a type of tumor cell death, and play a major role in stimulating the immune system in cancer therapy. These data indicate that **58**, either alone or in combination with etoposide, could induce immunogenic cell death (Fig. 9). Recently, Li et al. achieved the total synthesis of gitonin (**58**) for the first time from readily commercially available tigogenin and evaluated the cytotoxicity of gitonin and its structural analogs against A549, HepG-2, and MCF-7 cells [47]. The trisaccharide analog of gitonin exhibited cytotoxicity comparable to that of gitonin, indicating that the molecular structure of the branched tetrasaccharide of gitonin may not be essential for its cytotoxic activity.

**Fig. 10:**
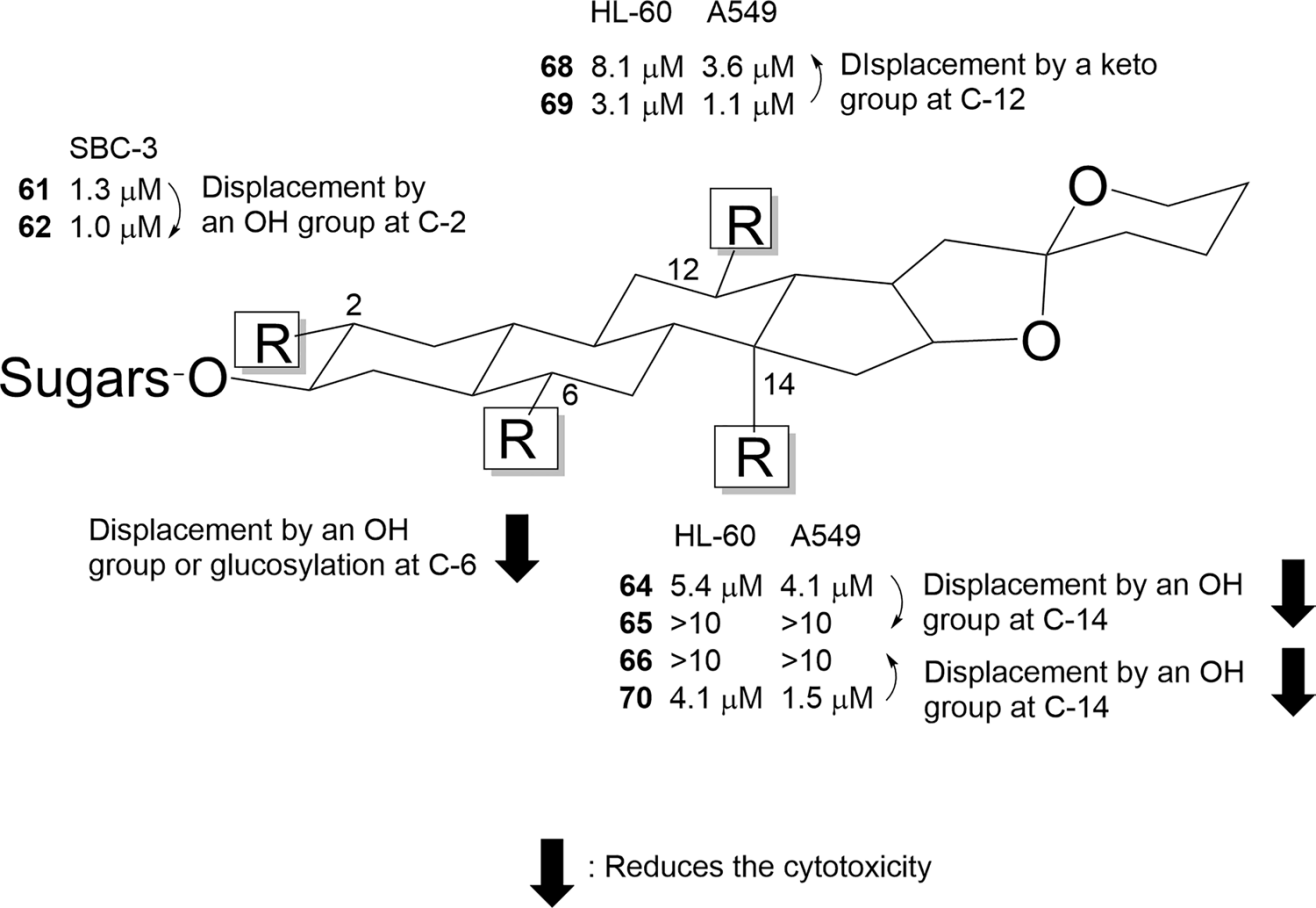
Structure–activity relationships of 5α-spirostanol glycosides. ➡: Reduces the cytotoxicity

Eight steroidal glycosides (**64**–**71**) isolated from the bulbs of *B. elegans* and *F. meleagris*, including five new compounds (**64**–**68**), were evaluated for their cytotoxic activities against HL-60 and A549 tumor cells, as well as TIG-3 normal cells [23, 24] (Fig. 8). Compounds **64**, **68**, and **69**–**70** showed cytotoxic activity against both HL-60 and A549 cells, demonstrating selective cytotoxicity toward tumor cells. Consistent with previous findings [48, 49], aglycones **64a**, **65a**, **67a**, **68a**, and **69a** lacked cytotoxic activity against all tested cell lines. Comparisons of their cytotoxic activities indicated that introducing a hydroxy group to the C-14α position reduced cytotoxicity (**64** vs. **65** and **66** vs. **70**), whereas the presence of a C-2α hydroxy group or C-12 ketone group did not affect cytotoxic activity (**61** vs. **62**, **68** vs. **69**). Preliminary analysis suggests that the presence of a C-6 hydroxy group or C-6 *O*-glucosyl group significantly reduces the cytotoxic activity of spirostanol glycosides [50, 51]. This study further supports the notion that the introduction of a hydroxy group to steroidal aglycones reduces their cytotoxic activity (Fig. 10).

### 5β and 5(6)-ene-spirostanol glycosides

**Fig. 11:**
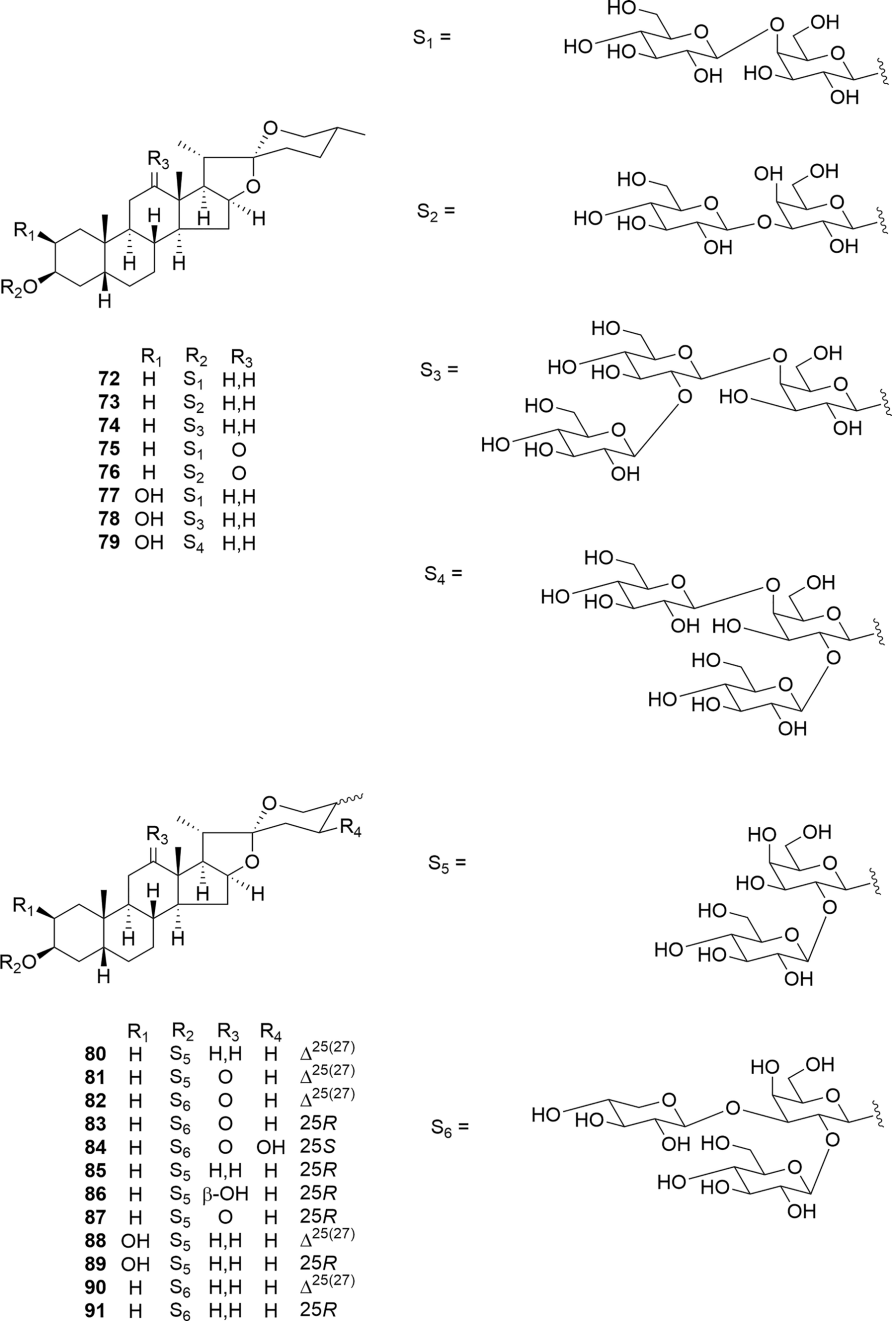
Structures of 5β-spirostanol glycosides **72**–**79** isolated from the whole plants of *Agave utahensis* and **80**–**91** isolated from the underground parts of *Yucca glauca*

*Agave utahensis* Engelm. and *Yucca glauca* Nutt. ex J. Fraser are members of the Agavaceae family. Twenty 5β-spirostanol glycosides (**72**–**91**), comprising thirteen undescribed compounds (**72**–**84**) were isolated from *A. utahensis* and *Y. glauca* (Fig. 11) [17, 29]. Compounds **72**–**79** were evaluated for their cytotoxic activity against HL-60 cells, whereas **80**–**91** were evaluated against A549 and HL-60 cells. Compounds **72**–**74**, **80**, **84**, **85**, and **88**–**91** exhibited cytotoxicity against HL-60 cells, whereas **80**, **85**, **90**, and **91** exhibited cytotoxicity against A549 cells. These findings suggest that the presence of C-2β hydroxy group, C-12 carbonyl group, and C-12 hydroxy group significantly diminishes the cytotoxic potential of these 5β-spirostanol glycosides. Compounds **72** and **80** induced apoptosis in HL-60 cells and conspicuously activated caspase-3.

**Fig. 12:**
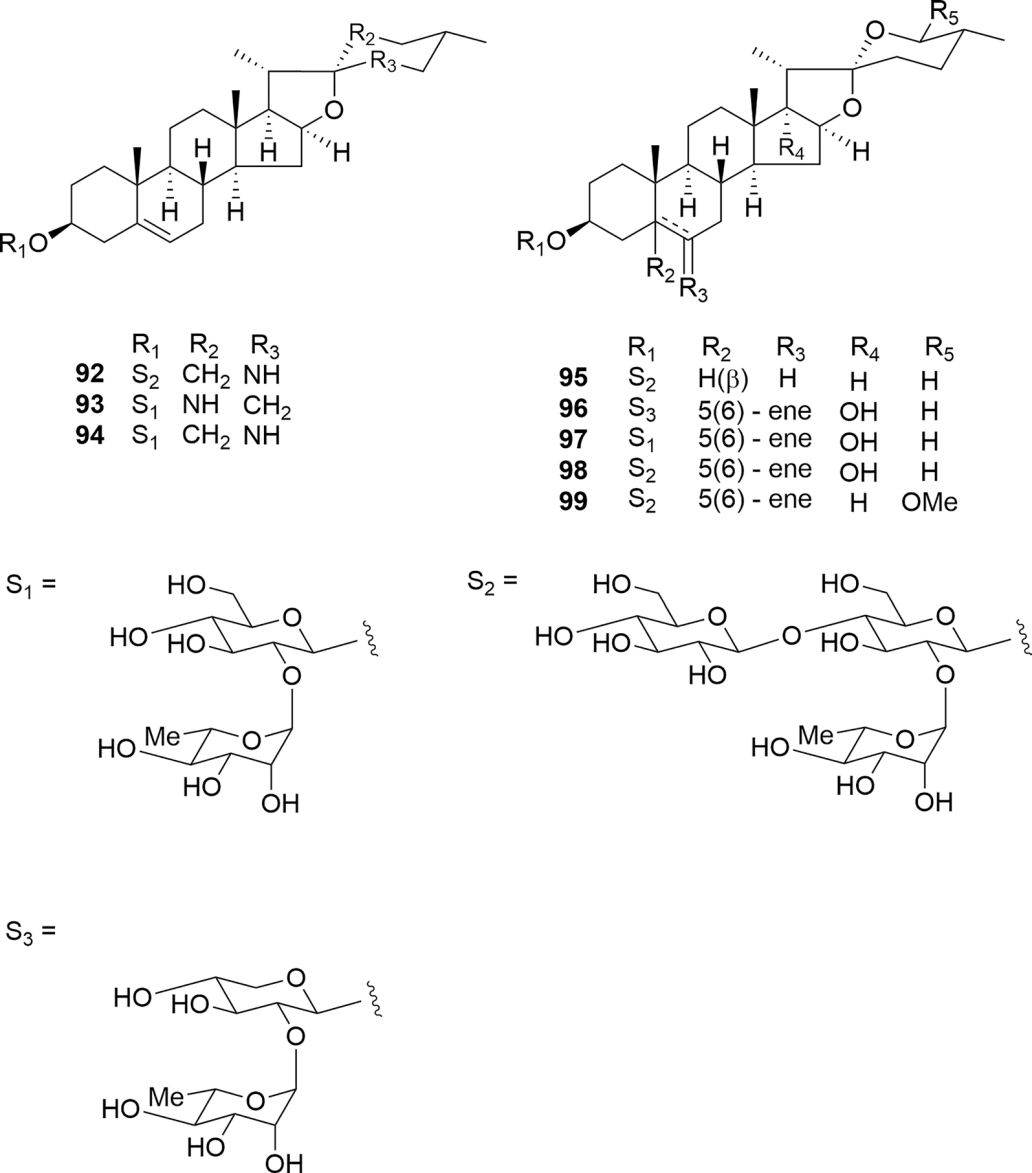
Structures of 5β and 5(6)ene-spirostanol glycosides **92**–**99** isolated from the bulbs of *Fritillaria meleagris*

5β and 5(6)-ene-spirostanol glycosides **92**–**99** were isolated from the bulbs of *F. meleagris*. The isolated compounds (**92**–**99**), including three novel compounds (**92**, **95**, and **96**), were evaluated for their cytotoxic activities against HL-60 and A549 cells (Fig. 12) [24, 32]. Among the steroidal alkaloids (**92**–**94**), only (22*S*)-spirosol glycosides **92** and **94** exhibited cytotoxic activity against HL-60 cells (IC_50_ 5.0 and 4.4 μM), whereas (22*R*)-spirosol glycoside (**93**) demonstrated selective cytotoxicity toward A549 cells (IC_50_ 7.9 μM) and induced apoptotic cell death without altering caspase-3 activity levels. Notably, **93** appears to induce apoptotic cell death in cultured tumor cells through distinct mechanisms of action. The absolute configuration of C-22 significantly influences the selective cytotoxicity of spirosol glycosides. The 5β-spirostanol glycoside (**95**) induced apoptotic cell death instead of cell cycle arrest in HL-60 cells in a time-dependent manner. Among the 5(6)-ene-spirostanol glycosides (**96**–**99**), neither **96** nor its aglycones (**92a**, **95a**, and **96a**) showed cytotoxicity against HL-60 and A549 cells. These results indicate that replacing the β-D-glucopyranosyl unit at C-3 of the aglycone with the β-D-xylopyranosyl unit reduced the cytotoxic activity.

**Fig. 13:**
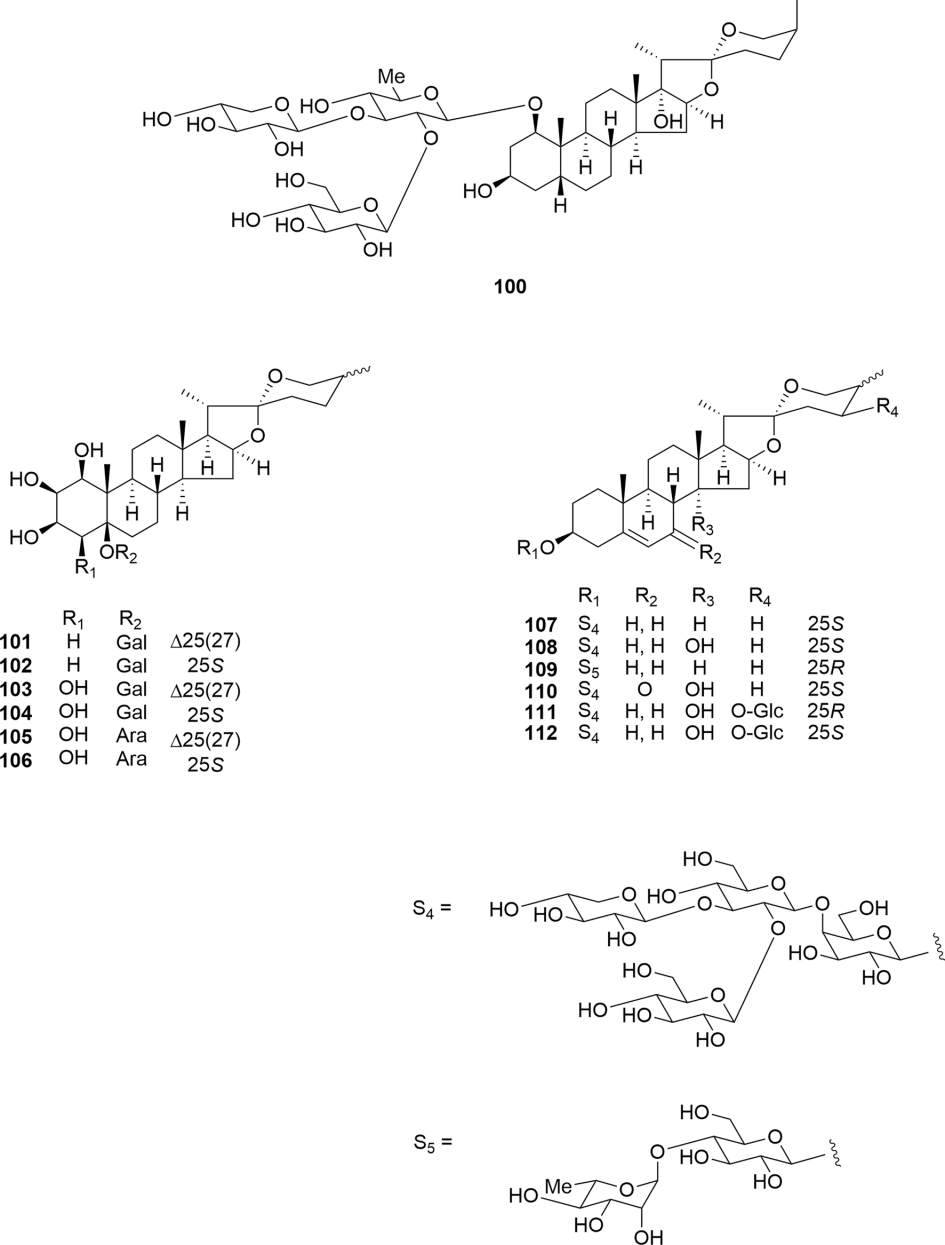
Structures of 5β and 5(6)ene-spirostanol glycosides **100**–**112** isolated from the rhizomes of *Convallaria majalis*

Convallasaponin A (**100**) and six 5β-polyhydroxylated spirostanol saponins (**101**–**106**) were isolated as novel compounds from *C. majalis* (Fig. 13). Dąbrowska-Balcerzak et al. also identified twelve 5β-polyhydroxylated spirostanol sapogenins and saponins from *C. majalis* [52]. Molecular docking simulations were conducted with the structures of the HER2 receptor and tubulin using in silico methods. Among these compounds, diols (less polar) exhibited higher affinities for the analyzed targets compared to tetrols and pentols (more polar), suggesting that the introduction of polar substituents to the steroidal nuclei reduced the binding effect [53, 54]. The 5(6)-ene-spirostanol glycosides (**107**–**112**), including three novel steroidal glycosides containing an *O*-β-D-glucopyranosyl-(1 → 2)-*O*-[β-D-xylopyranosyl-(1 → 3)]-*O*-β-D-glucopyranosyl-(1 → 4)-β-D-galactopyranose (lycotetrose) unit (**110**–**112**), were evaluated for their cytotoxic activity against HL-60, A549, HSC-4, and HSC-2 cells (Fig. 13) [14]. Compound 107 showed cytotoxic activity against all four tumor cell lines (IC_50_ 0.96–3.15 μM) and led to necrotic cell death in a dose-dependent manner. As we previously reported, the introduction of polar substituents to the steroidal nuclei resulted in reduced cytotoxicity (**107** vs. **108**, **110**–**112**).

## Furostanol glycosides and *pseudo*-furostanol glycosides

Furostanol glycosides have hemiacetal structure and pseudo-furostanol glycosides have Δ^20(22)^-unsaturation at C-22, respectively. Numerous phytochemical investigations and biological assays have been performed using spirostanol glycosides. However, the corresponding furostanol or Δ^20(22)^-pseudo-furostanol glycosides have received insufficient attention [53]. Here, we present our phytochemical study of furostanol and Δ^20(22)^-pseudo-furostanol glycosides to elucidate their cytotoxic constituents.

**Fig. 14:**
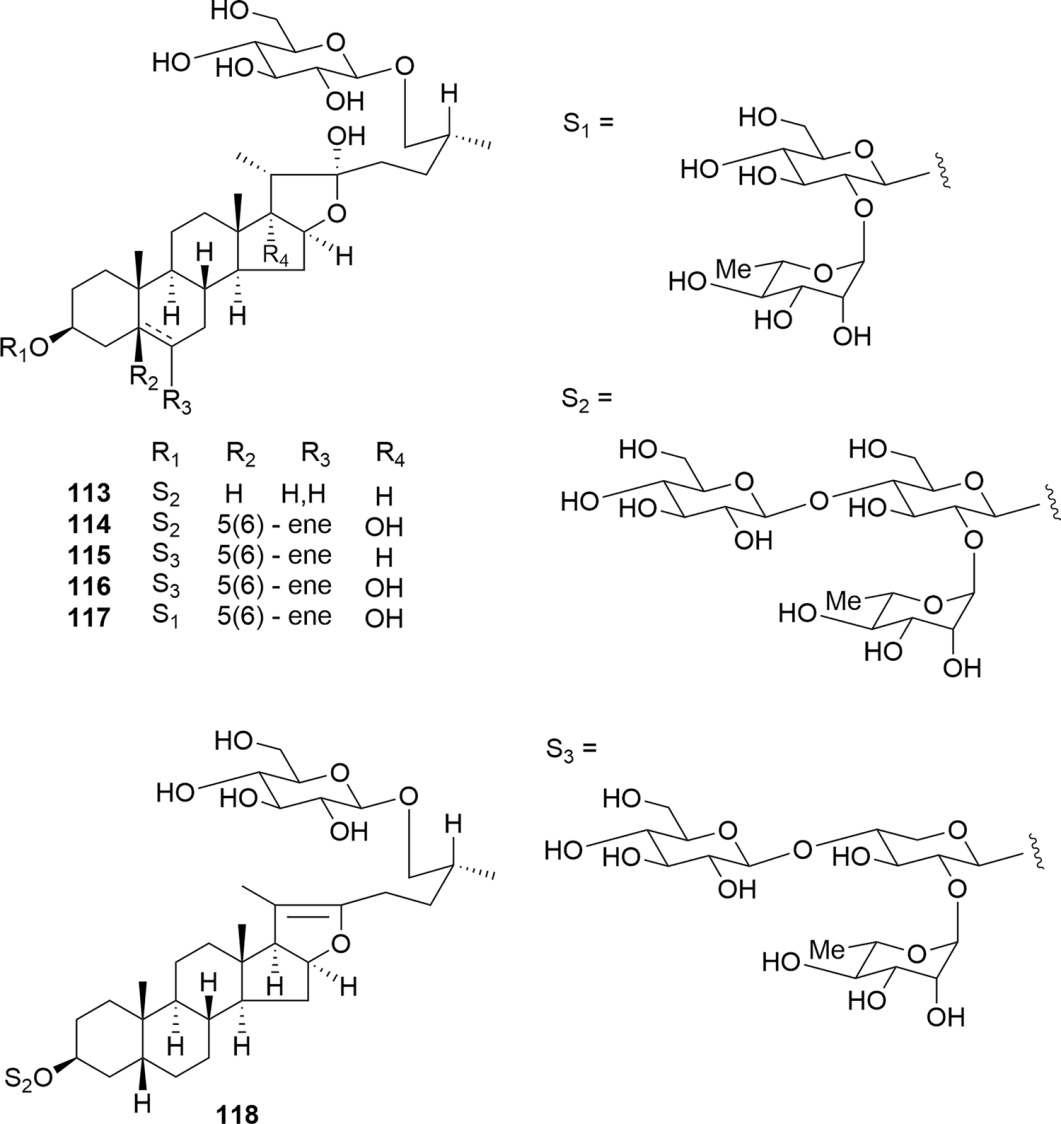
Structures of furostanol and pseudo-furostanol glycosides **113**–**118** isolated from *Fritillaria meleagris*

Compounds **113**–**118** isolated from the bulbs of *F. meleagris* were evaluated for cytotoxicity against HL-60 and A549 cells (Fig. 14) [24]. 5β-Furostanol glycoside **113** displayed cytotoxic activity against both tumor cell lines (IC_50_ 3.8 and 6.8 μM), whereas **114** and **118** were cytotoxic to A549 cells (IC_50_ 7.6 and 4.5μM). Notably, pseudo-furostanol glycoside **118** demonstrated cytotoxic activity. Our findings revealed that substituting the D-glucose unit at C-3 of the aglycone with a D-xylose unit attenuated the cytotoxicity of **114**.

**Fig. 15:**
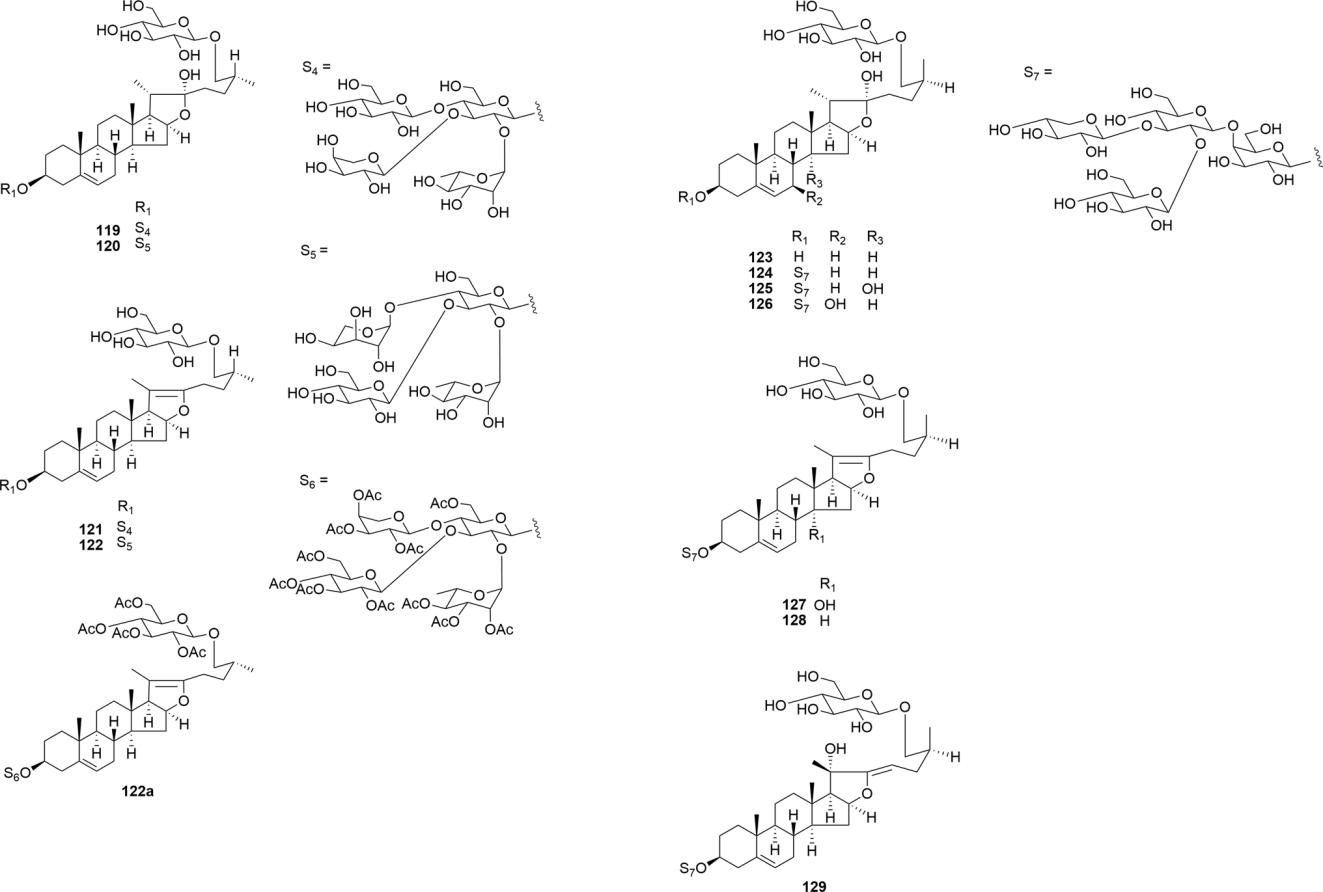
Structures of furostanol and pseudo-furostanol glycosides **119**–**122** from the bulbs of *Lilium pumilum* and **123**–**129** from the bulbs of *Convallaria majalis*

**Fig. 16:**
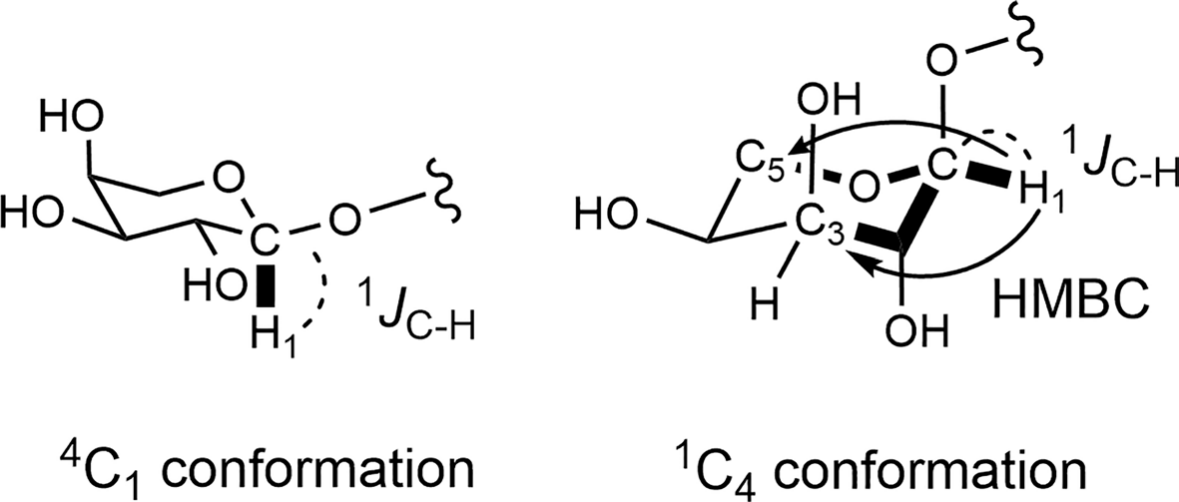
α-L-Arabinopyranosyl moieties as ^4^C_1_ conformation in **119** and **121** and^1^C_4_ conformation in **120** and **122**

Compounds **119**–**122**, which were isolated from the bulbs of *Lilium pumilum*, comprise rare types of steroidal glycosides featuring a 2,3,4-trisubstituted β-D-glucopyranosyl moiety at C-3 of the aglycone (Fig. 15) [30]. Typically, α-L-arabinopyranosyl groups adopt a stable ^4^C_1_ conformation in glycosides (**119** and **121**). However, when the C-2 position of the arabinopyranosyl group is substituted with a ^4^C_1_ glycosyl group, such as D-glucose, D-galactose, and D-xylose, it assumes a ^1^C_4_ conformation to mitigate the steric hindrance from these C-2 substituents (**120** and **122**). The small ^3^*J*_H-1,H-2_ coupling constant (3.2–3.4 Hz), large ^1^*J*_H-1,C-1_ value, and three-bond coupled strong HMBC correlations from the anomeric proton to the C-3 and C-5 carbons of the arabinopyranosyl moiety suggest the conformation of the L-arabinopyranosyl group as ^1^C_4_ with an α-orientation of the anomeric center (Fig. 16). Notably, the ^1^C_4_ α-L-arabinopyranosyl moiety in **122** converts to the ^4^C_1_ conformation on peracetylation (**122a**). This interesting steric behavior of arabinopyranose remains unexplained due to steric hindrance. Recent study reports indicate that the bulky disaccharide of the rhamnosyl-(1 → 2)-glucosyl group attached to C-3 of the inner xylosyl moiety forces the α-L-arabinosyl group linked to C-4 of the same xylosyl moiety to adopt a ^1^C_4_ conformation in triterpene glycosides [55]. Therefore, future studies should gather additional data. The isolated compounds **119**–**122** exhibited no cytotoxicity against HL-60 cells at sample concentrations up to 15 μM.

Compounds **123**–**129** were isolated from the bulbs of *C. majalis* and their cytotoxicity was evaluated (Fig. 15). Compound **124**, a furostanol lycotetroside of **107**, exhibited cytotoxicity exclusively toward adherent cell lines of A549, HSC-4, and HSC-2 cells (IC_50_ 2.97–11.04 μM), inducing apoptotic cell death in A549 cells via caspase-3/7 activity in a time-dependent manner [14]. The cytotoxicity mechanism predominantly involves 3-*O*-lycotetroside, with the introduction of polar substituents to the steroidal nuclei, diminishing the cytotoxic potential of furostanol and spirostanol glycosides [56].

**Fig. 17:**
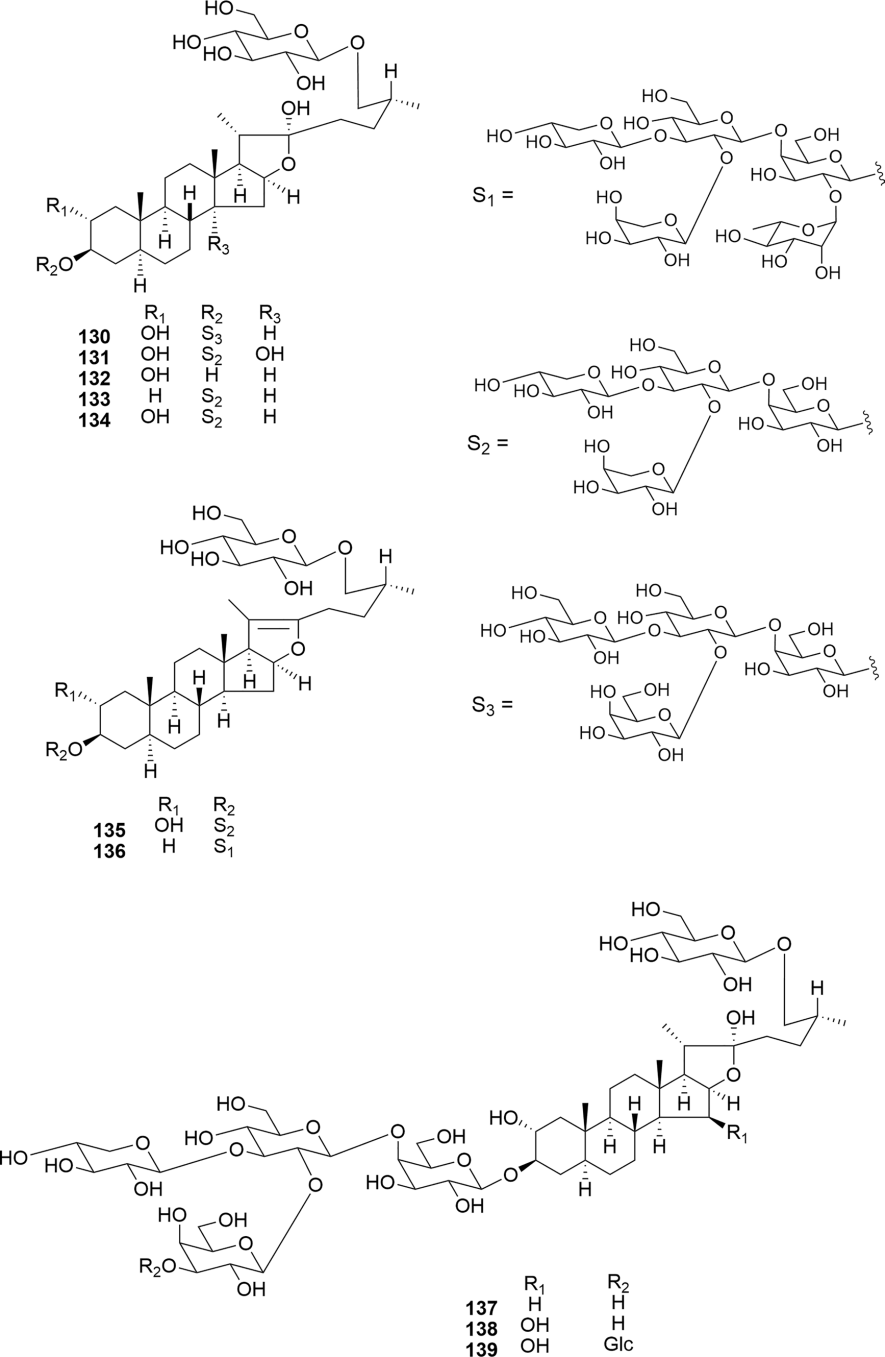
Structures of furostanol and pseudo-furostanol glycosides **130**–**136** from the bulbs of *Bessera elegans* and **137**–**139** from the seeds of* Digitalis purpurea*

Compounds **130**–**136** were isolated from the bulbs of *B. elegans* (Fig. 17). Compounds **130**, **133**, **134**, and **136** exhibited selective cytotoxicity toward HL-60 and A549 cells (IC_50_ 0.5–6.2 μM) while demonstrating no significant impact on cell growth in TIG-3 normal cells (IC_50_ > 10 μM) [23]. The findings suggest that the presence of a C-2α hydroxy group does not influence cytotoxic activity, whereas the introduction of a hydroxy group to the C-14α position decreases cytotoxicity. Notably, pseudo-furostanol glycoside **136** selectively inhibited tumor cell growth in a time-dependent manner and induced apoptosis in HL-60 and A549 cells. Furthermore, **136** induced cell cycle arrest at the G0/G1 phase in A549 cells.

Furostanol glycosides **137**–**139**, which were isolated from the seeds of *D. purpurea*, did not show cytotoxic activity against SBC-3 cells (IC_50_ > 10 μM) and their combination with etoposide did not demonstrate synergistic effects (Fig. 17) [26].

## Conclusions and discussions

We conducted chemical investigations of higher plants, focusing on steroidal glycosides. Our ongoing research has led to the discovery of several steroidal glycosides in ornamental garden plants and medicinal herbs that exhibit cytotoxic activity or possess novel skeletons. Screening and systematic chemical analysis of plant extracts are thus promising avenues for uncovering the structural diversity of steroidal glycoside, despite the time investment required. Structure–activity relationships (SAR) are frequently observed for spirostanol and furostanol glycosides. The introduction of polar substituents, such as hydroxy, carbonyl, and glucosyl groups, to the aglycone moiety has been observed to diminish cytotoxicity. Notably, the presence of a hydroxy group in the axial position of the aglycone moiety may influence the cytotoxicity. While replacement of the terminal sugar in the sugar sequence at the C-3β hydroxy group of the aglycone had minimal impact on activity, the inner sugar attached to C-3 of the aglycone played a significant role in cytotoxicity. In our study, steroidal glycosides demonstrated cytotoxicity against various tumor cells (A549, ACHN, HepG-2, HL-60, HSC-2, HSC-3, HSC-4, HSG, and SBC-3) through diverse mechanisms, including necrosis, caspase-dependent or -independent apoptosis, cell proliferation arrest, and the induction of DAMPs release. Notably, the spirostanol glycosides showed synergistic cytotoxicity when combined with etoposide, whereas this synergistic effect was not observed for the corresponding furostanol glycosides. The identification of potential novel anti-cancer agents requires precise SAR determination and in vivo evaluation of steroidal glycosides. In addition, cytotoxicity assays against normal cell lines have not been sufficiently reported, and the selectivity of steroidal glycosides toward cancer cells needs to be studied. Further detailed studies on the cytotoxicity mechanisms, such as autophagy and ferroptosis, molecular targets, and chemical structures, are warranted.
